# Th17/Treg ratio derived using DNA methylation analysis is associated with the late phase asthmatic response

**DOI:** 10.1186/1710-1492-10-32

**Published:** 2014-06-24

**Authors:** Amrit Singh, Masatsugu Yamamoto, Jian Ruan, Jung Young Choi, Gail M Gauvreau, Sven Olek, Ulrich Hoffmueller, Christopher Carlsten, J Mark FitzGerald, Louis-Philippe Boulet, Paul M O'Byrne, Scott J Tebbutt

**Affiliations:** 1 James Hogg Research Centre for Heart Lung Innovation, St. Paul’s Hospital, University of British Columbia, Vancouver, BC, Canada; 2 Institute for HEART + LUNG Health, Vancouver, BC, Canada; 3 Prevention of Organ Failure (PROOF) Centre of Excellence, Vancouver, BC, Canada; 4 Vancouver Coastal Health Research Institute, Vancouver General Hospital, Vancouver, BC, Canada; 5 Department of Medicine, Division of Respiratory Medicine, UBC, Vancouver, BC, Canada; 6 Department of Medicine, McMaster University, Hamilton, ON, Canada; 7 Epiontis GmbH, Berlin, Germany; 8 Quebec Heart and Lung Institute, Laval University, Québec City, QC, Canada

**Keywords:** Allergen inhalation challenge, Asthma, Asthmatic response, DNA methylation, Epigenetic cell counting, Peripheral blood, Th17/Treg ratio, nCounter Elements

## Abstract

**Background:**

The imbalance between Th17 and Treg cells has been studied in various diseases including allergic asthma but their roles have not been fully understood in the development of the late phase asthmatic response.

**Objectives:**

To determine changes in Th17 and Treg cell numbers between isolated early responders (ERs) and dual responders (DRs) undergoing allergen inhalation challenge. To identify gene expression profiles associated with Th17 and Treg cells.

**Methods:**

14 participants (8 ERs and 6 DRs) with mild allergic asthma underwent allergen inhalation challenge. Peripheral blood was collected prior to and 2 hours post allergen challenge. DNA methylation analysis was used to quantifiy the relative frequencies of Th17, Tregs, total B cells, and total T cells. Gene expression from whole blood was measured using microarrays. Technical replication of selected genes was performed using nanoString nCounter Elements.

**Results:**

The Th17/Treg ratio significantly increased in DRs compared to ERs post allergen challenge compared to pre-challenge. Genes significantly correlated to Th17 and Treg cell counts were inversely correlated with each other. Genes significantly correlated with Th17/Treg ratio included the cluster of genes of the leukocyte receptor complex located on chromosome 19q 13.4.

**Conclusions:**

Th17/Treg imbalance post-challenge may contribute to the development of the late phase inflammatory phenotype.

## Introduction

The imbalance between a proinflammatory T helper 17 (Th17) and a regulatory T (Treg) cell phenotype may play a crucial role in allergic airway inflammation [1]. Experimental models have shown that Th17 cells typically promote neutrophilic inflammation, and also play important roles in airway hyperresponsiveness in concert with Th2 cells [2]. In peripheral blood, Th17 cell counts have been shown to be higher in subjects with allergic asthma compared to healthy controls [3,4]. The percentage of Th17 cells and IL-17 levels in peripheral blood have been shown to be significantly elevated 24 hours after allergen challenge in dual responders compared to early responders or healthy controls [5]. On the other hand, Treg cells maintain immune homeostasis and regulate immune responses to allergens by preventing excessive inflammatory responses [6]. Treg cells were originally identified as CD4^+^CD25^+^ T cells with a function to suppress immune responses [7]. In order to identify Treg cells, *FOXP3* expression as a specific marker has been used, however, it is also expressed in activated non-suppressor T cells [8,9]. Low levels of the IL-7 receptor (CD127) in combination with high expression of CD4 and CD25 can be used to isolate highly purified suppressive Tregs [10]. Recently, DNA methylation analysis of the Treg specific demethylation region (TSDR) within the *FOXP3* locus has been used to enumerate Treg cells, [11] which have been shown to significantly correlate with CD4^+^CD25^+^CD127^lo^ and CD4^+^CD25^+^CD127^lo^FOXP3^+^ cells [12].

We have previously demonstrated that peripheral blood is a useful biological material with which to study changes in the blood transcriptome, proteome and metabolome of individuals with mild atopic asthma undergoing allergen inhalation challenge [13-16]. In the present study, we have used qPCR based DNA methylation analysis to estimate the number of Th17 cells, Treg cells, T cells and B cells in peripheral blood of mild atopic asthmatics undergoing allergen inhalation challenge. In the same individuals, we also analysed gene expression profiles in whole blood using microarrays to identify genes correlated with each cell type. We hypothesized that changes in specific immune cell counts in peripheral blood would be associated with the allergen-induced late phase asthmatic response.

## Methods

### Study participants and allergen inhalation challenge

The Institutional Review Boards of the participating institutions, University of British Columbia, McMaster University and Université Laval, approved this study. Fourteen individuals were recruited as part of the AllerGen NCE Clinical Investigator Collaborative (Canada) and provided written informed consent to undergo an allergen inhalation challenge. All participants were non-smokers, free of other lung diseases, and not pregnant. Diagnosis of asthma was based on the Global Initiative for Asthma criteria. Participants were diagnosed with mild allergic asthma, and only used intermittent short-acting bronchodilators for treatment of their asthma. Participants had a baseline FEV_1_ ≥ 70% of predicted, and the PC_20_, provocative concentration of methacholine required to produce a 20% decrease in FEV_1_, was ≤16 mg/mL.

Skin prick tests were used to determine allergies to cat, and the dose of cat allergen extract for inhalation. Methacholine and allergen challenges were conducted as triad visits. On the first and third day, participants underwent methacholine inhalation tests for assessments of airway hyperresponsiveness (AHR) as described previously [17,18]. The allergen-induced shift (post/pre in PC_20_) was evaluated as the change in AHR. On the second day participants underwent allergen inhalation challenge with extracts of cat pelt or hair in doubling doses until a drop in FEV_1_ of at least of 20% was achieved, then FEV_1_ was measured at regular intervals up to 7 hours post-challenge as described previously [19]. All participants developed an early response which resolved within 1–3 hours after challenge. Participants that demonstrated a maximum drop in FEV_1_ of greater than 15% between 3 to 7 hours after allergen inhalation were classified as dual responders (DRs). Participants having an FEV_1_ drop of 10% that was still falling at the end of the 7 hour observation period were categorized as DRs if they also demonstrated a drop in PC_20_ (post compared to pre methacholine challenge). Participants who showed neither a drop in FEV_1_ > 15% between 3 to 7 hours after challenge nor a decreased PC_20_ were classified as isolated early responders (ERs).

### Blood collection and isolation of RNA and DNA

Peripheral blood was obtained immediately before and 2 hours post-challenge in PAXgene Blood RNA tubes (PreAnalytiX, Qiagen/BD, Valencia, CA, USA) for RNA and in K2 EDTA Vacutainer tubes (BD, Franklin Lakes, NJ, USA) for buffy coat and complete blood count (CBC) measurements. Cellular RNA was purified from 2.5 mL of whole blood in PAXgene tubes according to the manufacturer’s protocols using the RNeasy Mini Kit (Qiagen, Chatsworth, CA, USA). Total DNA was isolated from whole blood or buffy coat from EDTA tubes using QIAamp DNA Blood Mini Kit (Qiagen) according to the manufacturer’s protocol.

### Epigenetic cell counting of lymphocyte subsets using DNA methylation analysis

Cell counting of lymphocyte subsets was performed by Epiontis (Berlin, Germany) using quantitative real-time PCR (qPCR) based DNA methylation analysis [20,21]. Briefly, bisulphite conversion [22] of genomic DNA resulting in either CpG-variants (if DNA is methylated) or TpG-variants (if DNA is unmethylated) was performed. Each qPCR assay is specific for either the demethylated *FOXP3* TSDR (for Tregs) or the demethylated *CD3D/G* (for T cells) or the demethylated *IL17A* (for Th17 cells) or the B cell specific demethylated gene region (for B cells) templates, since the demethylated version of these regions have been shown to be exclusively present in Treg, T cells, Th17 cells and B cells respectively. The other qPCR assay is specific for a control region within the *GAPDH* gene, a target that is demethylated in all cells. The *GAPDH* PCR assay serves as a “load control” as it estimates the number of “total cells” in a given sample. The percentage of Treg cells, T cells, Th17 cells and B cells in a sample is calculated as:

Percentage of a particular cell-type = [Copy Equivalents as determined with the PCR assay targeting the cell-specific DNA target region (e.g. TpG^TSDR^)]/[Copy Equivalents as determined with the *GAPDH* qPCR assay (TpG^GAPDH^)] × [100] × [2^**a)**^].

In the equation above, the “Copy Equivalents” as determined by the cell-specific PCR assay corresponds to “Treg cells”, or “T cells” or “Th17 cells” or “B cells” copies, respectively. The “Copy Equivalents” as determined with the *GAPDH* PCR assay corresponds to the “total cell” copies, respectively. A factor of “100” is used to translate the result into percentage of cells.

^a)^ Only for Tregs a factor of “2” is applied in the equation to correct for the fact that each cell has two copies of the (demethylated) *GAPDH* gene but each Treg has just one copy of the demethylated *FOXP3* gene. As *FOXP3* is X-chromosomally located, each Treg holds exactly one copy of the demethylated *FOXP3* gene. Tregs from male subjects hold one X chromosome on which the *FOXP3* gene is demethylated. In contrast, each Treg from a female subject has two X chromosomes (and thus two copies of the *FOXP3* gene) but one X chromosome is inactivated (i.e. fully methylated) and it exists as a Barr body in the cell.

### Microarray gene expression assay

Genome-wide expression profiling, labelling and array hybridization were performed using Affymetrix Human Gene 1.0 ST arrays (Affymetrix, Santa Clara, CA, USA). All microarray data has previously [16] been deposited into the Gene Expression Omnibus (GSE40240). All ‘CEL’ files were normalized using the Robust Multiarray Average (RMA).

### nCounter Elements

Technical replication of selected genes was performed using a new digital technology, nCounter Elements (NanoString, Seattle, USA). nCounter Elements allows users to combine nCounter Elements General Purpose Reagents (GPRs) with unlabelled probes that target specific genes of interest (http://www.nanostring.com/elements/). 100 ng of each RNA sample is added to the TagSet in hybridization buffer and incubated at 65°C for 16 hours. The TagSet consists of a reporter tag and capture tag that hybridize to the user designed gene-specific probe A and probe B complex. Automated processing per cartridge on the PrepStation (high sensitivity protocol) occurs for 3 hours. After a 2.5 hour scan per cartridge, counts are acquired from the GEN2 Digital Analyzer. Details regarding data normalization can be found in the supplementary material.

### Statistical and bioinformatics analysis

Linear models were used to test the association between immune cell frequencies and cell-specific gene expression profiles. Cell counts and all combinations of cell ratios (T, B, Treg and Th17) were compared using linear regression models. All microarray data were analysed using the linear models for microarrays (limma) R-library [23]. The Benjamini-Hochberg false discovery rate (FDR) was used to correct for multiple testing. Partial least squares (PLS), from the mixOmics R-library [24] was used to identify the relationship between cell-specific gene lists. Statistical analyses were performed in the statistical computing program R version 3.0.1 [25].

To test for the enrichment of gene lists, GeneGo network analysis was performed using MetaCore from Thomson Reuters. Network analyses were performed on gene lists created by ranking genes by the scores which rank the subnetworks to saturation with the objects from the initial gene list.

## Results

### Participant characteristics

The 14 participants were classified into eight isolated early responders (ERs) and six dual responders (DRs), as shown in Table 1. The mean drop in FEV_1_ during the late phase in DRs (21.3 ± 3.2) was 4 times greater (p < 0.05) compared to ERs (5.1 ± 1.4). Table 1 also shows that all participants exhibited an immediate drop in FEV_1_ of greater than 20%.

**Table 1:** Participant demographics

**Participant ID**	**Age (year)**	**Sex (M:F)**	**Allergen**	**Pre [PC**_**20**_**] (mg/mL)**	**Post [PC**_**20**_**] (mg/mL)**	**Allergen-induced shift**^**b**^	**% fall in FEV1**	
							**Early**	**Late**
**ER**								
1	28	F	Cat Pelt	12.8	ND	ND	20.3	4.8
2	34	F	Cat Pelt	2.8	6.1	2.3	21	1.5
3	27	M	Cat Pelt	4.5	1.8	0.39	34.4	0
4	42	F	Cat Hair	5.3	8.6	1.6	42.1	11.1
5	29	F	Cat Pelt	0.4	ND	ND	44.3	0
6	31	M	Cat Pelt	11.8	16	1.4	24.2	7.5
7	28	F	Cat Hair	9.4	16	1.7	27.1	7.1
8	42	M	Cat	0.1	ND	ND	23	9
Mean ± SE	32.6 ± 2.2	3:5		2.8^a^	7.5^a^	1.5 ± 0.3	29.6 ± 3.2	5.1 ± 1.4
**DR**								
9	23	F	Cat Hair	0.3	0.2	0.60	38.9	31.8
10	26	F	Cat Hair	5.1	1.5	0.30	31.4	14.9
11	49	F	Cat Hair	3.6	1.0	0.27	25.3	12.6
12	26	M	Cat Hair	0.9	1.0	1.1	31.5	15.6
13	27	F	Cat Pelt	0.6	0.1	0.19	48.3	25.8
14	52	F	Cat	ND	ND	ND	33	27
Mean ± SE	33.8 ± 3.3	1:5		1.3^a^	0.5^a^	0.5 ± 0.1	34.7 ± 3.2	21.3 ± 3.2^c^

ND - Not determined; all participants were challenged with cat allergen.

^a^geometric mean (PC_20_ values are measured on a log scale).

^b^[PC_20_]_post_/[PC_20_]_pre_.

^c^p < 0.05 versus ER group.

### Correlation between immune cell frequency and cell-specific gene expression

**Figure 1:**
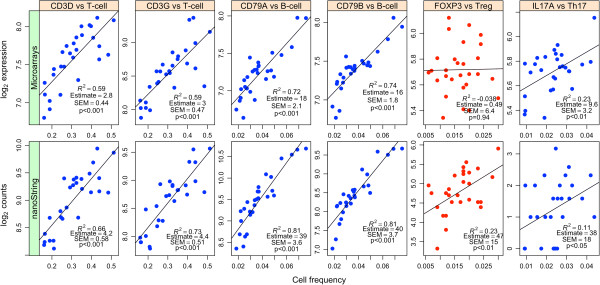
**Scatter plots of immune cells quantified using DNA methylation analysis with the corresponding cell-specific gene expressions profiles.** x-axis: relative cell-type frequencies of T, B, Treg and Th17 cells in whole blood; y-axis: a) top row: gene (*CD3D*, *CD3G*, *CD79A*, *CD79A*, *FOXP3*, and *IL17A*) expression intensities measured using microarrays and b) bottom row: gene expression counts measured using nCounter Elements from nanoString.

Sum of the T cell and B cell frequencies obtained using the methylation assays strongly correlate (Spearman r = 0.95) with the lymphocyte frequency obtained using a hematolyzer (Additional file 1: Figure S1). T cell, B cell and Th17 cell counts were significantly positively correlated with the genes targeted in epigenetic cell counting in both the microarray (Figure 1; top row) and nanoString (Figure 1; bottom row) platforms. Treg cell counts were not correlated with *FOXP3* gene expression measured using microarrays but was significantly correlated using nanoString, suggesting greater sensitivity of the platform (Figure 1, red points).

### Th17 to Treg ratio discriminates early from dual responders after challenge

**Figure 2:**
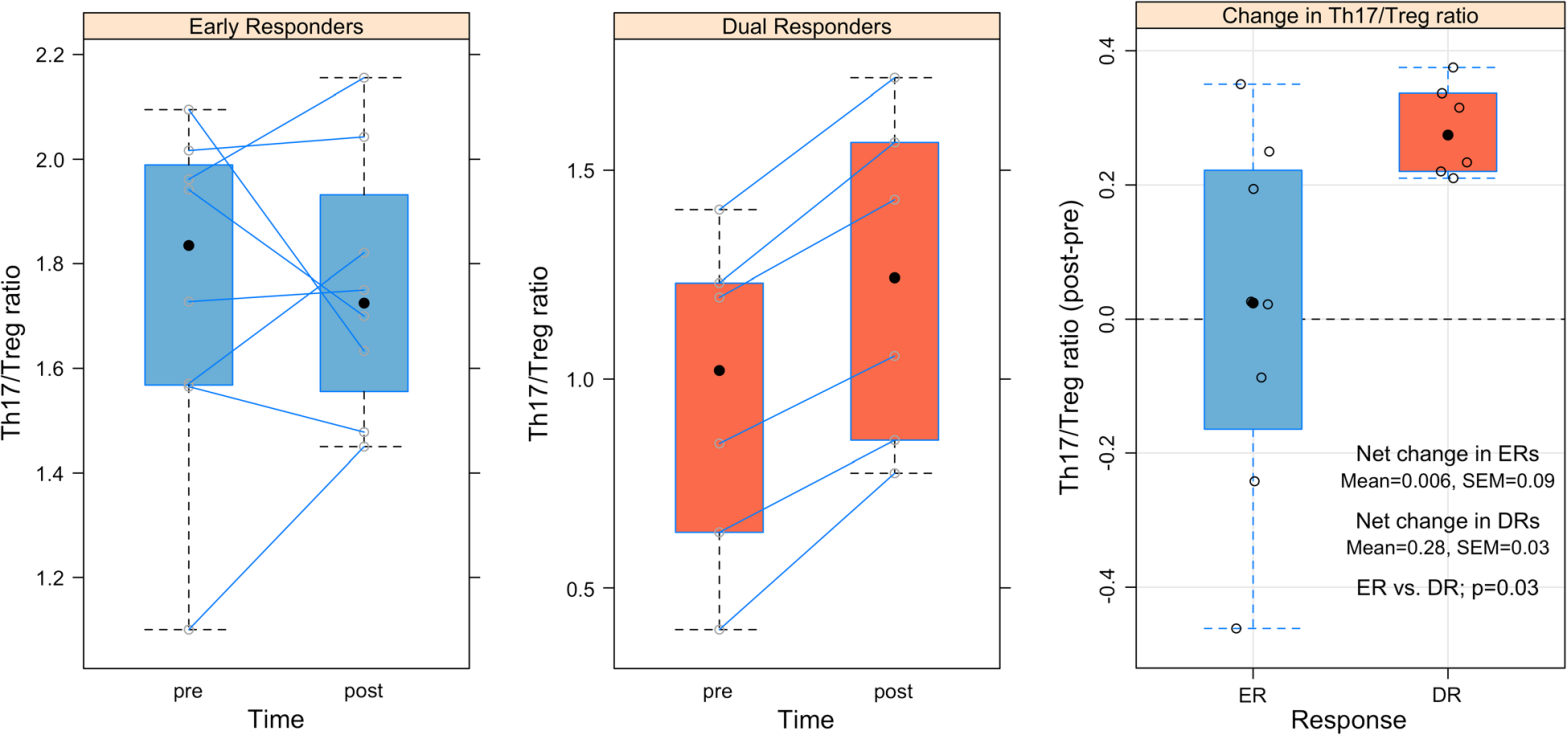
**Change in the Th17/Treg ratio in early and dual responders from pre to post challenge.** Th17/Treg ratio in ERs **(left panel)** and DRs **(middle panel)** at pre and post-challenge. The change in the Th17/Treg ratio (post-pre) in ERs and DRs **(right panel)**. Solid black points depict the median value of the data for each boxplot.

Allergen inhalation did not significantly change T cell, B cell, Treg cell and Th17 cell counts in either ERs or DRs. In addition, comparing the change in cell counts in ERs with the change in cell counts in DRs (ΔER vs. ΔDR) no significant cell-types were identified (Table 2A). Next, the ratios between different cell-types were analyzed (Table 2B). Table 2B shows that the Th17/Treg ratio significantly (p = 0.03) increased in DRs compared to ERs, from pre to post challenge. Figure 2 shows that the Th17/Treg ratio did not change from pre to post challenge in ERs (net change = 0.006 ± 0.09), whereas the Th17/Treg ratio increased in DRs (net change = 0.28 ± 0.03).

**Table 2:** Comparing immune cell-frequencies and cell/cell ratios between early and dual responders after allergen challenge

**A. Comparing immune cell-frequencies between ERs and DRs after allergen challenge**			
	**Fold-change (post-pre) in early responders**	**Fold-change (post-pre) in dual responders**	Δ**ER vs. **Δ**DR**
	**Mean ± SEM**	**Mean ± SEM**	**p-value**
T cell	-2.20 ± 1.40	-4.26 ± 2.39	0.45
B cell	-0.20 ± 0.17	-0.11 ± 0.26	0.77
Th17 cell	-0.22 ± 0.21	0.14 ± 0.16	0.22
Treg cell	-0.12 ± 0.06	-0.42 ± 0.17	0.10
**B. Comparing immune cell/cell ratios between ERs and DRs after allergen challenge**			
	**Fold-change (post-pre) in early responders**	**Fold-change (post-pre) in dual responders**	Δ**ER vs. **Δ**DR**
	**Mean ± SEM**	**Mean ± SEM**	**p-value**
T/B	-0.59 ± 0.70	-0.74 ± 0.43	0.87
Th17/T	0.002 ± 0.006	0.009 ± 0.002	0.31
Treg/T	-0.0008 ± 0.005	-0.006 ± 0.002	0.38
B/Th17	-0.009 ± 0.10	-0.37 ± 0.21	0.13
B/Treg	-0.002 ± 0.22	0.42 ± 0.18	0.18
Th17/Treg	0.006 ± 0.09	0.28 ± 0.03	0.03

### Genes associated with Th17 and Treg cells

**Figure 3:**
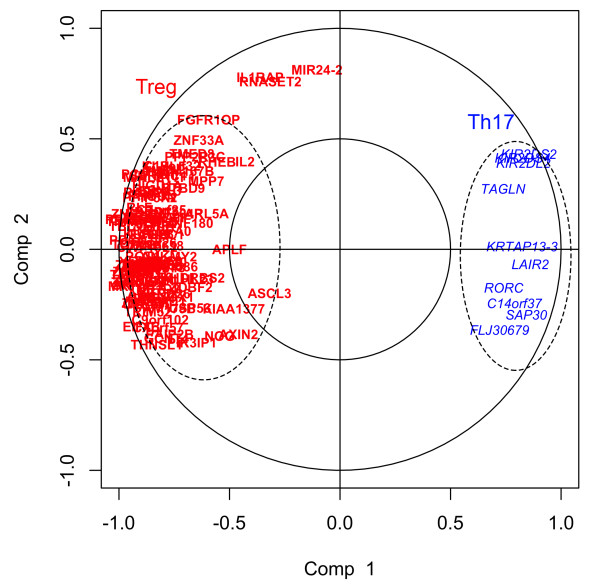
**Correlation circle depicting the strength of correlation between Treg genes (red) and Th17 genes (blue) with their respective latent variables (Comp 1 and Comp 2).** The Treg genes (red) show a strong negative correlation with the Th17 genes (blue). Vectors drawn from the origin to each of the points (genes) allows one to determine the relationship between genes: 1) if the angle between two vectors is less than 90°, there exists a positive correlation between the two genes, 2) if the angle between two vectors is greater than 90°, there exists a negative correlation between the two genes, and 3) if the angle between two vectors is equal to 90°, the correlation between the two genes is zero.

A multiple linear regression model (limma) was used to identify genes whose expression levels correlated with the frequencies of specific cell-types independent of changes in the frequencies of other cell-types (gene expression ~ Th17 + Treg + B-cells + other T-cells, where other T-cells = overall T-cells minus Th17 and Treg). 10 (99) genes were positively correlated with Th17 (Treg) cells at an FDR of 10%, with no overlapping genes between the two lists. Th17 genes included *KIR2DS2*, *TAGLN*, *C14orf37*, *KRTAP13-3*, *SAP30*, *KIR2DS4*, *LAIR2*, *FLJ30679*, *RORC* and *KIR2DL2*. The 99 Treg genes were enriched (FDR = 5%) for 27 pathways including many relevant regulatory pathways such as IL-2 regulation of translation, Regulation of telomere length and cellular immortalization, Regulation of T cell function by CTLA-4 (Additional file 1: Figure S2). Partial Least Squares (PLS) was used to determine the correlation between the set of 10 Th17 genes and the set of 99 Treg genes. Figure 3 depicts the results of PLS using a correlation circle (see Gonzalez et al. [26] for complete details on graphical outputs of PLS). Vectors drawn from the origin to each of the points (genes) allows one to determine the relationship between genes: 1) if the angle between two vectors is less than 90°, there exists a positive correlation between the two genes, 2) if the angle between two vectors is greater than 90°, there exists a negative correlation between the two genes, and 3) if the angle between two vectors is equal to 90°, the correlation between the two genes is zero. Figure 3 shows that the Th17 genes were inversely correlated with Treg genes (angle greater than 90°).

### Genes significantly correlated with the Th17/Treg ratio

**Figure 4:**
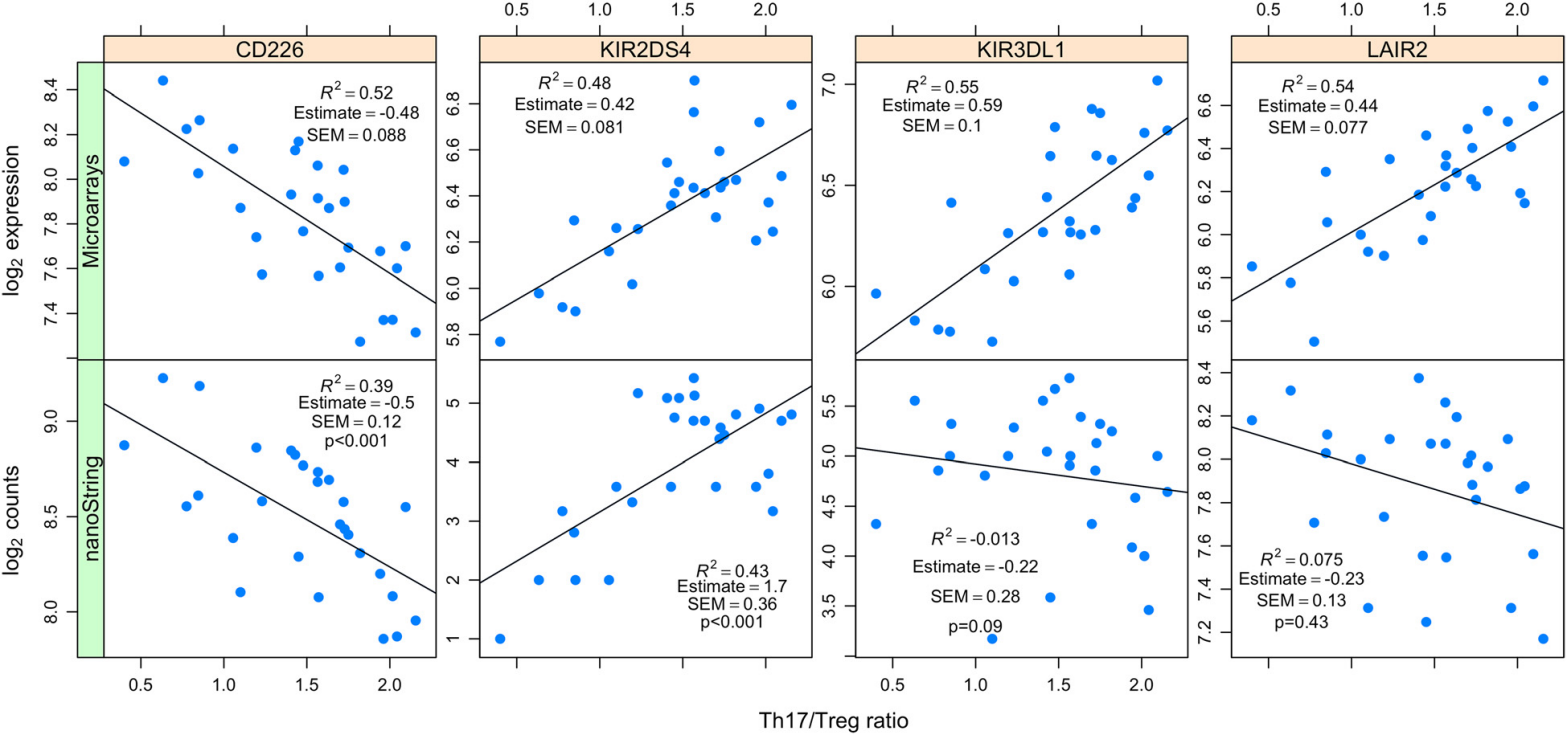
**Scatter plots of genes significantly correlated with the Th17/Treg ratio.** x-axis: Th17/Treg ratio; y-axis: a) top row: gene (*CD226*, *KIR2DS4*, *KIR3DL1*, and *LAIR2*) expression intensities measured using microarrays and b) bottom row: gene expression counts measured using nCounter Elements from nanoString.

To investigate the relationship of Th17/Treg ratio and gene expression profiles, we identified correlated genes in the entire sample set. We identified 13 genes significantly correlated with Th17/Treg ratio using limma (FDR = 5%, Table 3). Interestingly, 7 genes (*KIR3DL1*, *LAIR2*, *KIR2DS2*, *KIR2DL2*, *CD226*, *KIR2DS4*, *KIR2DS1*) belong to the leukocyte receptor complex (LRC) located on chromosome 19q13.4, and were shown to be positively correlated except *CD226*. However, of the four genes profiled using nanoString, only *CD226* and *KIR2DS4* successfully replicated (Figure 4). The top-listed transcriptional network in GeneGo network analysis for the 13 significant genes included regulatory functions in immune responses (Additional file 1: Table S1).

**Table 3:** Genes significantly correlated to Th17/Treg ratio in Pearson tests (FDR <0.05)

**Gene**	**r**	**p value**	**FDR**
*TAGLN*	0.78	7.59E-07	0.02
*C14orf37*	0.77	3.16E-06	0.02
KIR3DL1*	0.75	3.82E-06	0.02
LAIR2*	0.75	4.80E-06	0.02
*CDCP1*	0.74	5.77E-06	0.02
KIR2DS2*	0.74	6.19E-06	0.02
*SAP30*	0.74	7.09E-06	0.02
KIR2DL2*	0.73	8.51E-06	0.02
CD226*	-0.73	9.83E-06	0.02
*ZNF286B*	-0.73	9.27E-06	0.02
*KRTAP13-3*	0.72	1.57E-05	0.03
KIR2DS4*	0.71	2.38E-05	0.043
KIR2DS1*	0.70	2.86E-05	0.048

*genes belonging to leukocyte receptor complex (LRC).

### Significantly different genes between ERs and DRs in Th17 or Treg

In a secondary analysis, we also analysed gene-cell correlations that significantly differed between early and dual responders, irrespective of allergen exposure, using limma. In GeneGo network analysis, genes differentially associated with Th17 (165 genes differentially associated with Th17 between ERs and DRs, p < 0.01) were enriched for immunological processes including immunoglobulin mediated immune response and adaptive immune response. Genes differentially associated with Treg between ER and DR (554 genes, p < 0.01) were enriched for immune processes. Although the genes differentially associated with Th17 cells between ERs and DRs did not achieve a stringent threshold of FDR, the top three genes, *S100B*, *MILR1* and *CHI3L1* (p-value < 0.001, FDR = 0.79, Additional file 1: Figure S3), have previously been reported to be involved in allergy or asthma [27-29]. Additional file 1: Figure S3 shows that all three genes were differentially correlated with Th17 cell counts with respect to the response class using both the microarray and nanoString platforms.

## Discussion

Although Th17 and Treg cells arise from a common precursor cell [30] they have opposing inflammatory roles which has been demonstrated in the context of autoimmune disease [31], infection [32], and recently allergic airway inflammation [1]. In the present study we demonstrate a potential Th17/Treg homeostatic imbalance using peripheral blood of isolated early and dual asthmatic responders (ERs and DRs) undergoing allergen inhalation challenge.

DNA methylation analysis used to enumerate various immune cells revealed good correlation with the cell-specific gene expression profiles as measured using microarrays. Technical replication using nCounter Elements from nanoString, a more sensitive platform indicated that *FOXP3* expression was indeed correlated with Treg cell counts. As a marker for human Tregs, however, *FOXP3* expression is of doubtful value, due to its transient expression in activated non-regulatory effector T cells [21]. In addition, other cell-surface markers such as CD127 or CD45RA have been used to isolate FOXP3^+^ Treg cell populations with high efficiency [33,34]. Epigenetic enumeration of Treg cells in the present study has been shown to positively correlate with CD4^+^CD25^+^CD127^lo^, and CD4^+^CD25^+^CD127^lo^FOXP3^+^[12] and thus are truly representative of suppressive Tregs.

The percentage of Treg cells did not significantly change in either ERs (-0.12 ± 0.06; p = 0.11) or DRs (-0.42 ± 0.17; p = 0.054), two hours post-challenge. Previous studies have also not shown significant changes in Treg cells in peripheral blood in DRs undergoing allergen inhalation challenge [35,36]. This may be due to many factors such as the time of the post-challenge blood draw, the cell-surface markers used to isolate the Treg cells as well as the small sample sizes (n = 6-11) used in these studies. Similarly, the percentage of Th17 cells also did not significantly change in ERs (-0.22 ± 0.21; p = 0.30) or DRs (0.14 ± 0.16; p = 0.44), after allergen challenge. Th17 cells have been shown to be increased 7 and 24 hours post-challenge in both ERs and DRs and the increase in DRs was greater than in ERs 24 hours post challenge [5]. Th17 cells as well as the concentrations of IL-17 and IL-22, have also been shown to be increased with the severity of allergic asthma [37]. Genes significantly positively correlated with Th17 cells included *RORC*, the transcription factor involved in Th17 differentiation, whereas genes significantly positively correlated with Treg genes was enriched for regulatory functions. Furthermore, Th17 and Treg cell associated genes were inversely correlated with each other, further implicating the phenotypic roles of these cell-types in allergic asthma.

Although neither cell-type significantly changed pre to post challenge, the change in the Th17/Treg ratio from pre to post challenge significantly (p = 0.03) differed between ERs and DRs. The Th17/Treg ratio increased in DRs whereas little change occurs in ERs after challenge. The increase in the Th17/Treg ratio in DRs is driven by an increase in the number of Th17 cells (0.14 ± 0.16) and a decrease in the number of Treg cells (-0.42 ± 0.17) due to allergen exposure. A possible mechanism of Th17/Treg imbalance was suggested by the genes that were correlated with Th17/Treg ratio. LRC on chromosome 19q13.4 encodes immunoglobulin super family receptors including killer immunoglobulin like receptors (KIRs) expressed on hematopoietic cells. Almost all LRC significant genes were positively correlated to Th17/Treg, whilst *CD226* is the only LRC gene negatively correlated. A previous study on differential expression of LRC genes revealed that KIRs and inhibitory receptor ILT2/LIR1 were expressed in activated T cells and that KIR levels in T cells are associated with resistance to activation-induced cell death [38]. These may suggest a new hypothesis that LRC gene expression patterns might be related to Th17/Treg ratio and involved in immune responses to inhaled allergen in asthmatics.

The statistical interaction analyses suggested differences in gene expression profiles in Th17 or Tregs between ERs and DRs. Interestingly, top-listed differentially expressed Th17-associated genes *S100B*, *MILR1* and *CHI3L1* have been reported to play roles in allergy and asthma. S100B^+^ lymphocytes in blood have been reported to consist of two subtypes; a cytotoxic T cell and a NK subtype [27]. In connection with the significant correlations between Th17 and KIR family, Th17 measured by epigenetic cell counting for *IL17A *might be related to other types of immune cells. This is supported by reports showing that IL-17 genes are expressed in non-CD4^+^ T cells such as γδ T cells, NK cells and Type 3 innate lymphoid cells, suggesting that innate immunity might be responsible for initiating this type of inflammation commonly associated with Th17 immunity [39,40]. Further studies are needed to clarify the disparity between true Th17 and IL17A-demethylated cells. *MILR1* is the gene for allergin-1 protein, which was recently identified to play an inhibitory role in mast cell functions [28]. Polymorphisms in *CHI3L1* as well as the concentration of its corresponding protein YKL-40 in serum has been associated with asthma and pulmonary function [29]. Our findings suggest that Th17 cell gene expression profiles are divergent between asthmatic responses and that these profiles might be related to immune mechanisms.

A limitation of this study is its small sample size, which reduces the statistical power in identifying true positives. Therefore we deemed a technical validation using a highly sensitive platform appropriate for this study. Independent replication will be important as part of future studies with larger sample sizes. Another limitation of the present study is that only a limited number of cell-types were studied using DNA methylation analysis, whereas quantification of a wide array of cell-types such as Th1, Th2, and Th9 cells would provide deeper biological insights into the mechanisms of allergic asthmatic responses. DNA methylation based qPCR assays for these cell-types will allow for tissue samples to remain unperturbed, and additional sources of variability, such as those observed in fluorescence activated cell sorting to be avoided.

The careful phenotyping of our participants, together with innovative epigenetic- and gene expression-based methodologies, have nevertheless revealed interesting directions for further investigations using large sample sizes and different allergens.

## Availability of supporting data

Supplementary Tables and Figures are shown in “Additional Documentation”.

## Abbreviations

Th17: T helper 17; Treg: Regulatory T; FOXP3: Forkhead box protein 3; TSDR: Treg specific demethylation region; ER: Isolated early responder; DR: Dual responder; Limma: Linear models for microarrays; PLS: Partial least squares; LRC: Leukocyte receptor complex.

## Competing interests

The authors declare that they have no competing interests.

## Authors’ contributions

AS and MY contributed equally to this work. GMG, PMO, CC, JMF, LPB, SJT participated in research design and provision of samples. MY, JR, SJT participated in the sample processing and following experiments. SO and UH performed the epigenetic cell counting assay. AS, MY, JYC, SJT conducted data analyses. AS, MY, CC, SJT participated in the writing of the paper. All authors read and approved the final manuscript.

## Supplementary Material

Additional file 1: Figure S1 Epigenetic vs. hematolyzer correlation of relative lymphocyte counts. **Figure S2.** Pathway analysis of 99 genes positively significantly associated with Treg cells. Only pathway with FDR<5% are shown. **Figure S3.** Correlation between genes and Th17 cells that differ between early and dual responders. **Table S1.** Top-listed transcription regulation network in GeneGo analysis for the genes significantly correlated to Th17/Treg ratio (FDR < 0.05). Click here for file
