# Supplementary material for: Th17/Treg ratio derived using DNA methylation analysis is associated with the late phase asthmatic response
Source: Allergy Asthma Clin Immunol. 2014 Jun 24;10(1):32. doi: 10.1186/1710-1492-10-32 (PMC4078401; doi:10.1186/1710-1492-10-32)
Supplement: Additional file 1: Figure S1 — Epigenetic vs. hematolyzer correlation of relative lymphocyte counts. Figure S2. Pathway analysis of 99 genes positively significantly associated with Treg cells. Only pathway with FDR<5% are shown. Figure S3. Correlation between genes and Th17 cells that differ between early and dual responders. Table S1. Top-listed transcription regulation network in GeneGo analysis for the genes significantly correlated to Th17/Treg ratio (FDR < 0.05). [file 1710-1492-10-32-S1.doc]

Supplemental Material

# Th17/Treg ratio derived using DNA methylation analysis is associated with the late phase asthmatic response

**Masatsugu Yamamoto, MD, PhD,^1,2,3,4*^ Amrit Singh, BSc,^1,2,5*^ Jian Ruan, MD, PhD,^1,2,5^ Jung Young Choi, BSc,^1,2^ Gail M. Gauvreau, PhD,^6^ Sven Olek, PhD,^7^ Ulrich Hoffmueller, PhD,^7^**

**Christopher Carlsten MD, MPH,^1,2,3,4^ J. Mark FitzGerald, MB, MD, MRCPI, FRCPC,^2,3,4^**

**Louis-Philippe Boulet, MD,^8^ Paul M. O'Byrne, MD, FRCPI,^6^ Scott J. Tebbutt, PhD,^1,2,3,5§^**

^1^James Hogg Research Centre, St. Paul’s Hospital, University of British Columbia, Vancouver, British Columbia, Canada

^2^Institute for HEART + LUNG Health, Vancouver, British Columbia, Canada

^3^Department of Medicine, Division of Respiratory Medicine, UBC, Vancouver, British Columbia, Canada; Canada

^4^Vancouver Coastal Health Research Institute, Vancouver General Hospital, Vancouver, British Columbia, Canada

^5^Networks of Centres of Excellence for Commercialization and Research (NCE CECR) Prevention of Organ Failure (PROOF) Centre of Excellence, Vancouver, British Columbia, Canada

^6^Department of Medicine, McMaster University, Hamilton, Ontario, Canada

^7^Epiontis GmbH, Berlin, Germany

^8^Quebec Heart and Lung Institute, Laval University, Québec City, Québec, Canada

*MY and AS contributed equally to this work

^§^Corresponding author

nanoString data normalization

1. The data is initially normalized to 8 positive spike-in controls in order to account for technical variability between assays. The geometric mean of the 8 positive controls is calculated for each sample. The normalization factor is calculated by dividing the geometric mean of the positive controls in each assay by the mean of the geometric means across all assays. All features for a given assay are multiplied by the positive control normalization factor.
2. The data is then normalized to 5 selected house-keeping genes; ACTB, FTL, GUSB, OAZ1 and UBC in order to account for RNA sample input variability between assays. The geometric mean of the 5 house-keeping controls is calculated for each sample. The normalization factor is calculated by dividing the geometric mean of the house-keeping controls in each assay by the mean of the geometric means across all assays. All features for a given assay are multiplied by the house-keeping control normalization factor.

Addtional file 1. Figure S1. Epigenetic vs. hematolyzer correlation of relative lymphocyte counts.


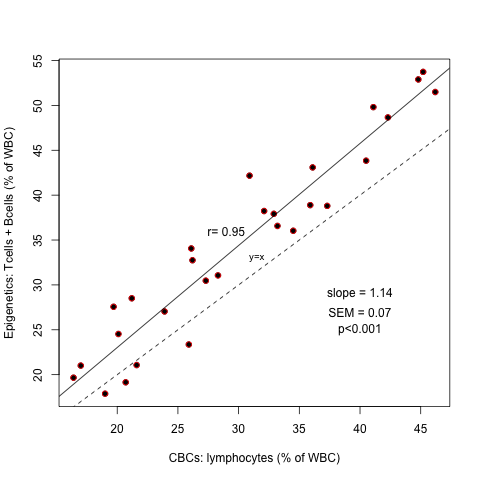


Addtional file 1. Figure S2: Pathway analysis of 99 genes positively significantly associated with Treg cells. Only pathway with FDR<5% are shown.
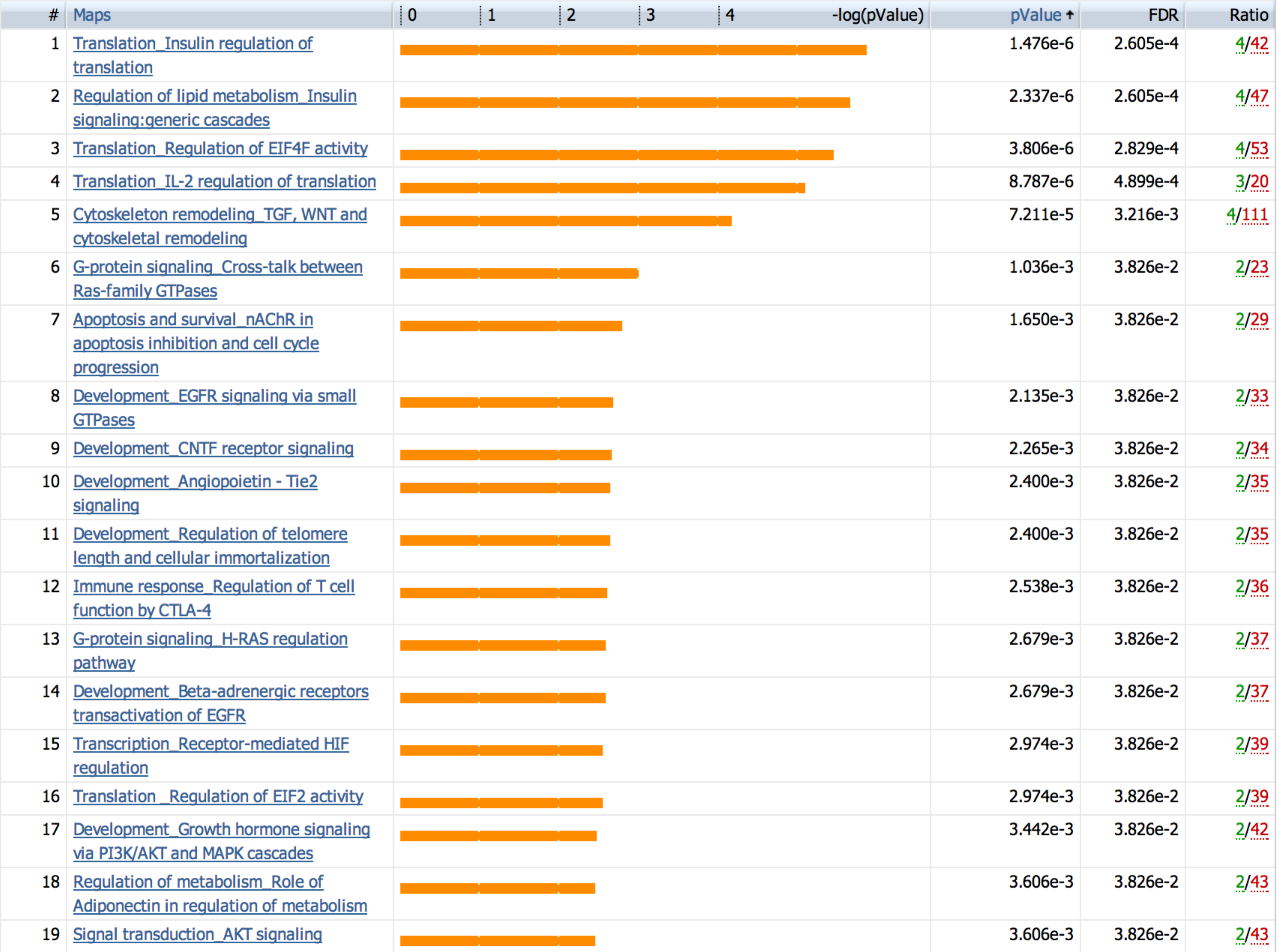


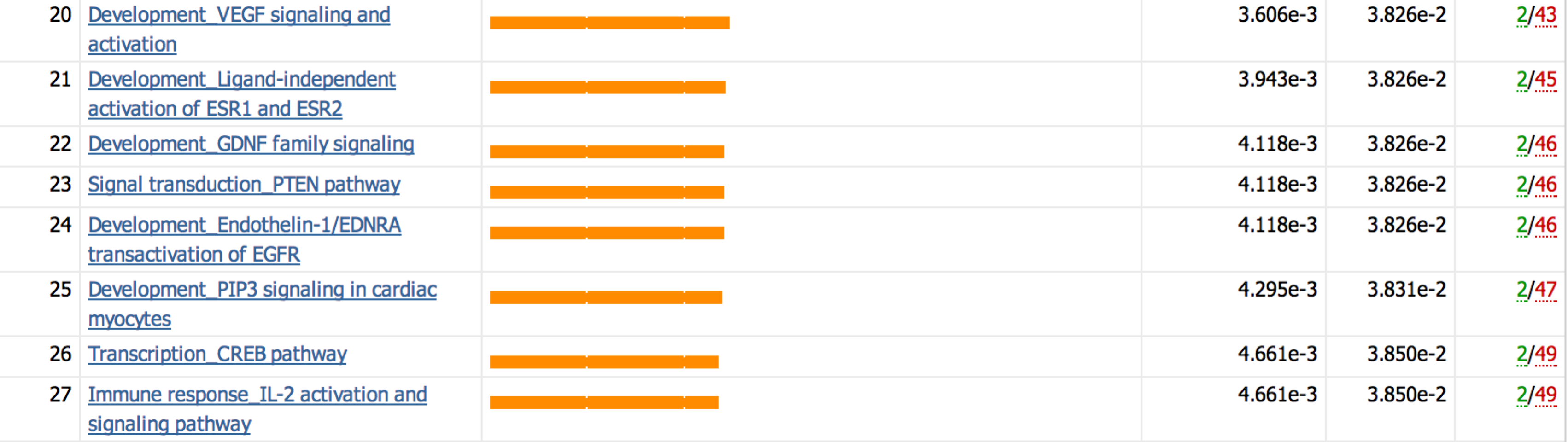


Addtional file 1. Figure S3: Correlation between genes and Th17 cells that differ between early and dual responders.

|  | S100B | MILR1 | CHI3L1 |
| --- | --- | --- | --- |
| Microarrays | 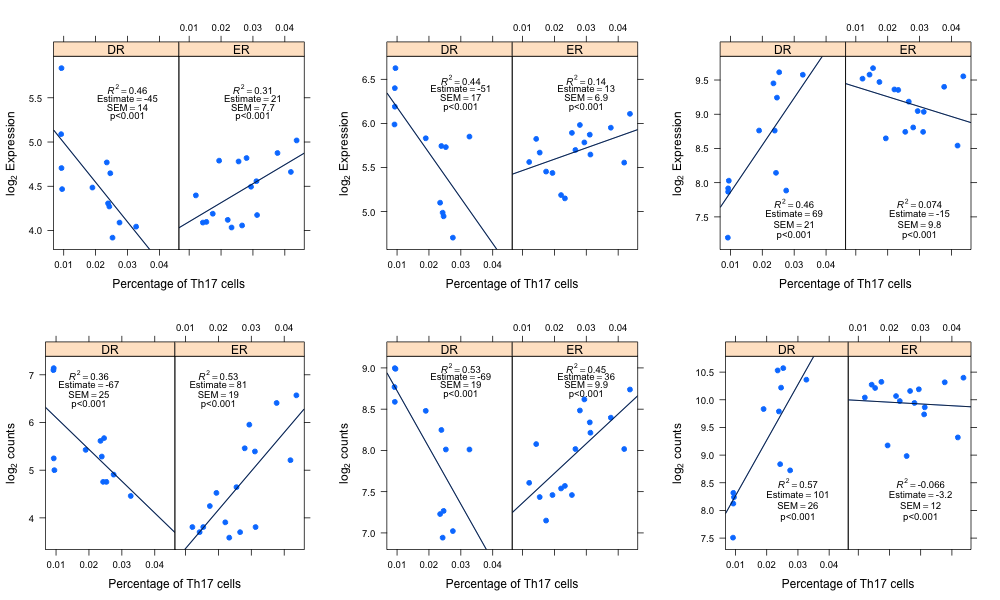 | 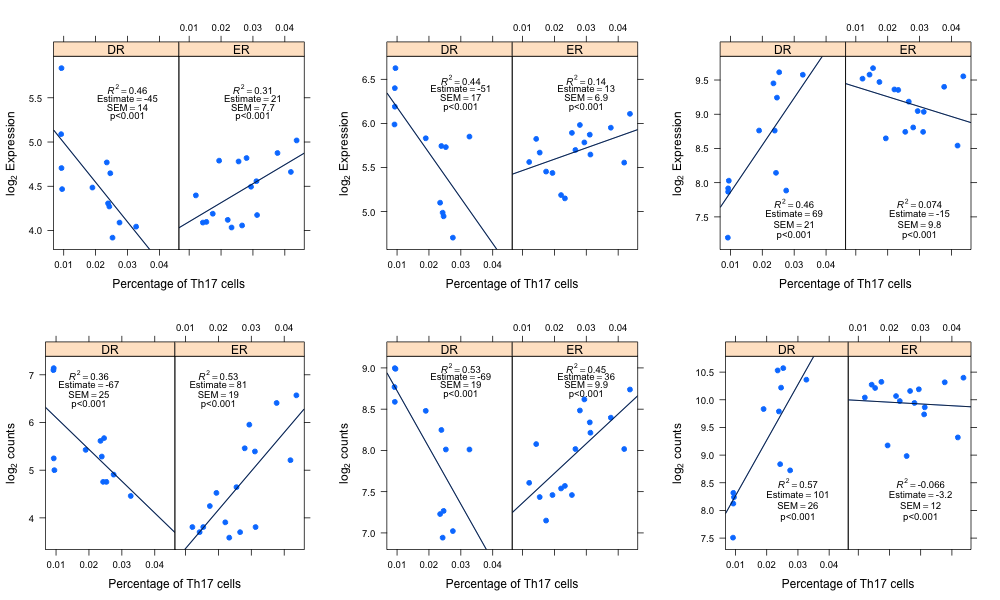 | 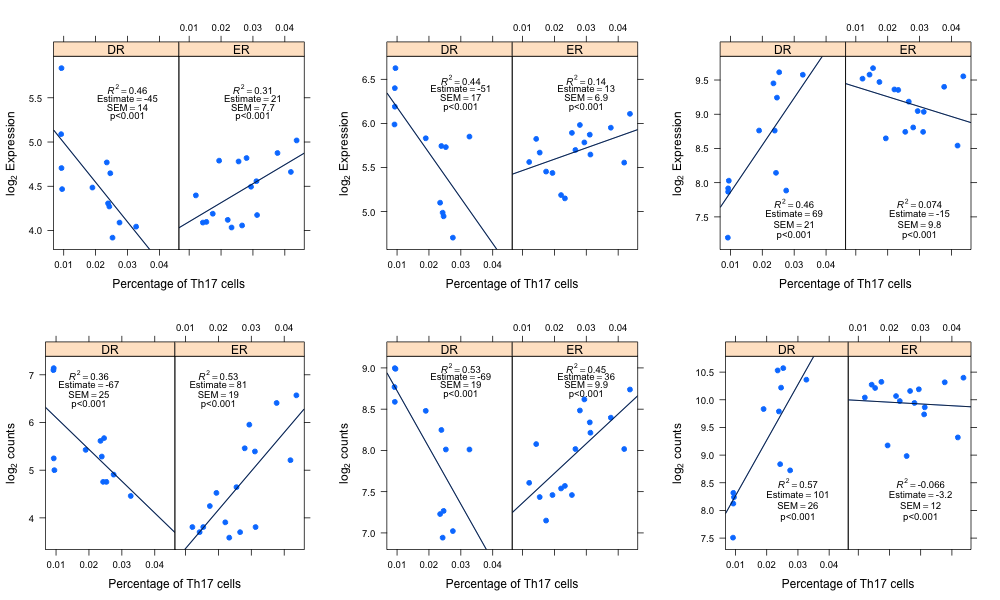 |
| nanoString | 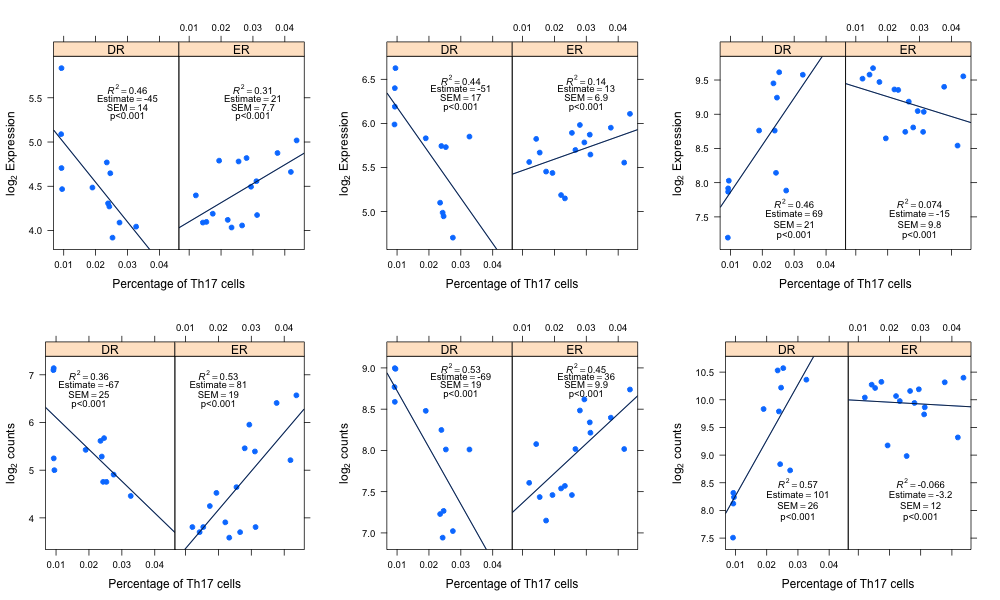 | 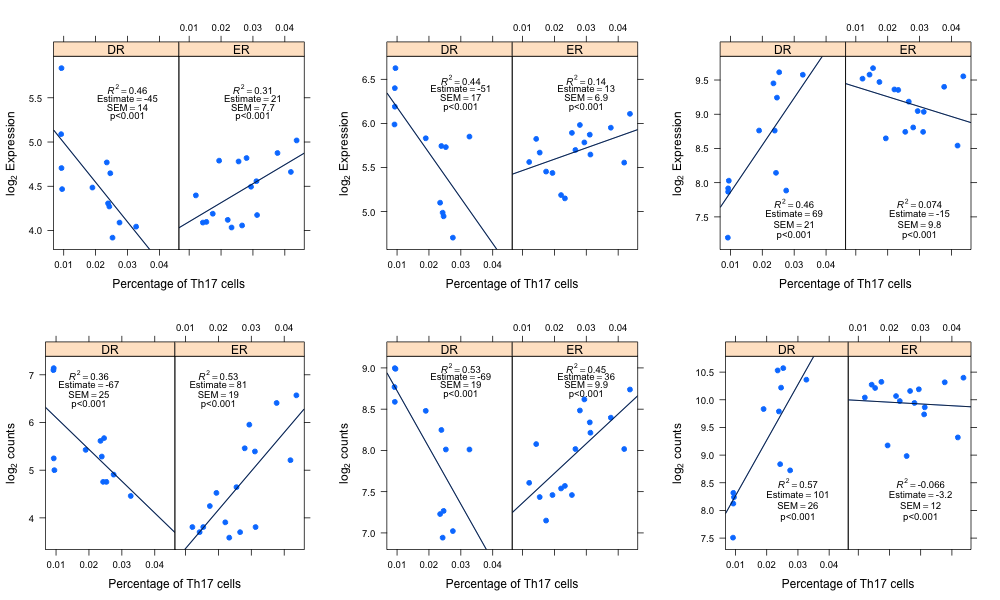 | 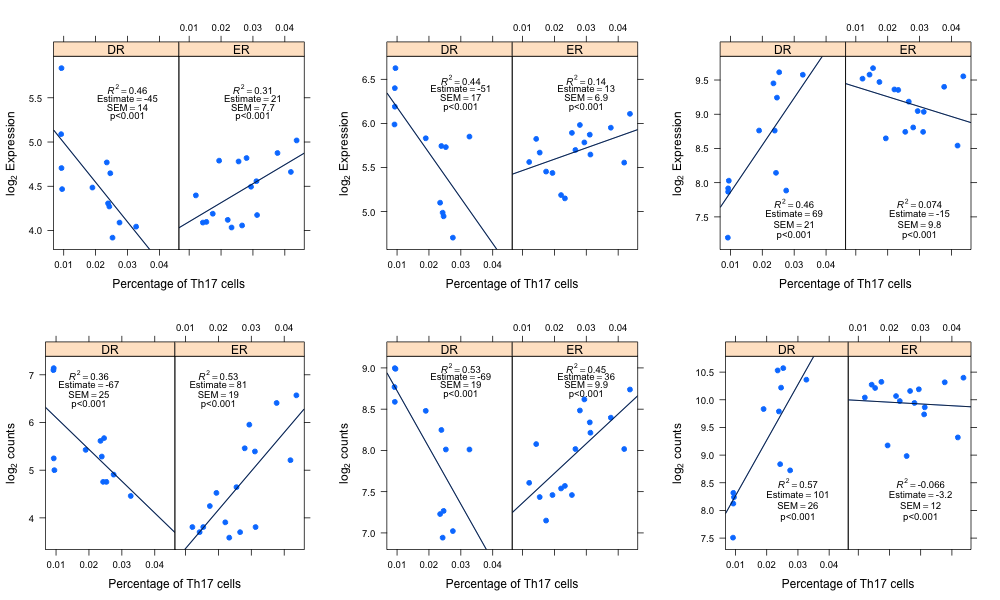 |

Addtional file 1. Table S1: Top-listed transcription regulation network in GeneGo analysis for the genes significantly correlated to Th17/Treg ratio (FDR < 0.05).

| **#** | **Network** | **GO processes** | **Total nodes** | **Seed nodes** | **p-Value** | **zScore** | **gScore** |
| --- | --- | --- | --- | --- | --- | --- | --- |
| 1 | SP1 | regulation of immune response (83.3%; 2.913e-07), regulation of immune system process (83.3%; 2.755e-06), regulation of response to stimulus (83.3%; 2.188e-04), positive regulation of Fc receptor mediated stimulatory signaling pathway (16.7%; 8.026e-04), immune system process (66.7%; 1.043e-03) | 7 | 6 | 9.930E-23 | 160.42 | 160.42 |
| 2 | AP-1 | regulation of immune response (100.0%; 1.726e-09), regulation of immune system process (100.0%; 2.615e-08), regulation of response to stimulus (100.0%; 5.431e-06), immune response (66.7%; 9.995e-05), positive regulation of Fc receptor mediated stimulatory signaling pathway (16.7%; 8.026e-04) | 7 | 6 | 9.930E-23 | 160.42 | 160.42 |
| 3 | ETS1 | regulation of immune system process (100.0%; 2.615e-08), regulation of immune response (83.3%; 2.913e-07), immune response (66.7%; 9.995e-05), regulation of response to stimulus (83.3%; 2.188e-04), positive regulation of Fc receptor mediated stimulatory signaling pathway (16.7%; 8.026e-04) | 7 | 6 | 9.930E-23 | 160.42 | 160.42 |
| 4 | CREB1 | regulation of immune response (100.0%; 4.998e-08), regulation of immune system process (100.0%; 4.809e-07), immune response (80.0%; 3.475e-05), regulation of response to stimulus (100.0%; 4.099e-05), immune system process (80.0%; 3.760e-04) | 6 | 5 | 7.300E-19 | 144.39 | 144.39 |
| 5 | AML1 (RUNX1) | regulation of immune system process (100.0%; 4.809e-07), regulation of immune response (80.0%; 7.030e-06), regulation of response to stimulus (100.0%; 4.099e-05), immune system process (80.0%; 3.760e-04), immune response (60.0%; 1.293e-03) | 6 | 5 | 7.300E-19 | 144.39 | 144.39 |
| 6 | c-Myc | regulation of metanephric cap mesenchymal cell proliferation (33.3%; 1.338e-04), positive regulation of metanephric cap mesenchymal cell proliferation (33.3%; 1.338e-04), fibroblast apoptotic process (33.3%; 1.338e-04), regulation of G2 phase of mitotic cell cycle (33.3%; 2.676e-04), negative regulation of G2 phase of mitotic cell cycle (33.3%; 2.676e-04) | 4 | 3 | 2.440E-11 | 106.1 | 106.1 |
| 7 | FOXP3 | regulation of immunoglobulin mediated immune response (66.7%; 1.580e-05), regulation of B cell mediated immunity (66.7%; 1.642e-05), positive regulation of lymphocyte mediated immunity (66.7%; 5.656e-05), positive regulation of leukocyte mediated immunity (66.7%; 5.656e-05), positive regulation of adaptive immune response based on somatic recombination of immune receptors built from immunoglobulin superfamily domains (66.7%; 6.129e-05) | 3 | 2 | 1.100E-07 | 81.67 | 81.67 |
| 8 | HNF1-alpha | renal glucose absorption (33.3%; 2.676e-04), regulation of pronephros size (33.3%; 4.013e-04), pronephros morphogenesis (33.3%; 5.351e-04), glucose import (33.3%; 9.363e-04), regulation of kidney size (33.3%; 1.070e-03) | 3 | 2 | 1.100E-07 | 81.67 | 81.67 |
| 9 | GCR-alpha | glucocorticoid mediated signaling pathway (33.3%; 4.013e-04), positive regulation of glucocorticoid receptor signaling pathway (33.3%; 4.013e-04), regulation of glucocorticoid receptor signaling pathway (33.3%; 6.688e-04), cellular response to magnesium ion (33.3%; 9.363e-04), regulation of glucocorticoid biosynthetic process (33.3%; 1.204e-03) | 3 | 2 | 1.100E-07 | 81.67 | 81.67 |
| 10 | HNF4-alpha | negative regulation of tyrosine phosphorylation of Stat5 protein (33.3%; 5.351e-04), regulation of microvillus assembly (33.3%; 5.351e-04), regulation of microvillus organization (33.3%; 6.688e-04), negative regulation of protein import into nucleus, translocation (33.3%; 6.688e-04), positive regulation of cholesterol homeostasis (33.3%; 8.026e-04) | 3 | 2 | 1.100E-07 | 81.67 | 81.67 |
